# RUO_2_ nanoparticle-decorated MWCNTS synthesized using a sonochemical method as reinforcing agents for PEI composite membranes

**DOI:** 10.1039/d4ra07606k

**Published:** 2024-12-16

**Authors:** S. I. Voicu, E. Vasile, A. Palla-Papavlu, R. Oprea, M. Ionita, A. M. Pandele

**Affiliations:** a Advanced Polymers Materials Group, National University of Science and Technology POLITEHNICA Bucharest 011061 Bucharest Romania madalina.pandele@upb.pub.ro pandele.m.a@gmail.com; b Faculty of Chemical Engineering and Biotechnology, National University of Science and Technology POLITEHNICA Bucharest 011061 Bucharest Romania; c National University of Science and Technology POLITEHNICA Bucharest Splaiul Independentei 313 060042 Bucharest Romania; d National Institute for Laser, Plasma and Radiation Physics Str. Atomistilor 409 Magurele 077125 Romania

## Abstract

This work presents a new and facile synthesis approach for multiwalled carbon nanotubes (MWCNTs) decorated with ruthenium oxide (RuO_2_) nanoparticles using a simple and efficient sonochemical method. The strong interaction and homogenous distribution of RuO_2_ nanoparticles on the surface of MWCNTs were revealed by Raman spectroscopy and transmission electron microscopy. The presence of metal oxide nanoparticles anchored onto the surface of MWCNTs was further confirmed by X-ray diffraction and energy-dispersive X-ray analysis. Furthermore, the chemical state of the MWCNTs before and after decoration with RuO_2_ was revealed by X-ray photoelectron spectroscopy. Scanning electron microscopy illustrated that the decoration process did not induce any modification on the morphology of the surface of MWCNTs. The percentage of RuO_2_ nanoparticles anchored onto the MWCNT surface was determined by thermogravimetric analysis, where 15% could be calculated considering the weight loss. Furthermore, both MWCNTs and decorated MWCNTs with RuO_2_ nanoparticles were used as nanofillers to develop some composite membranes using polyetherimine as polymer matrices. The morphological and structural properties of the membranes were characterized by SEM and XRD. The mechanical properties of the composite membranes, contact angle and antimicrobial properties using *Escherichia coli* and *Staphylococcus aureus* were also studied.

## Abstract 2

This work presents a new and facile synthesis approach for multiwalled carbon nanotubes (MWCNTs) decorated with ruthenium oxide (RuO_2_) nanoparticles using a simple and efficient sonochemical method.

## Graphical abstract

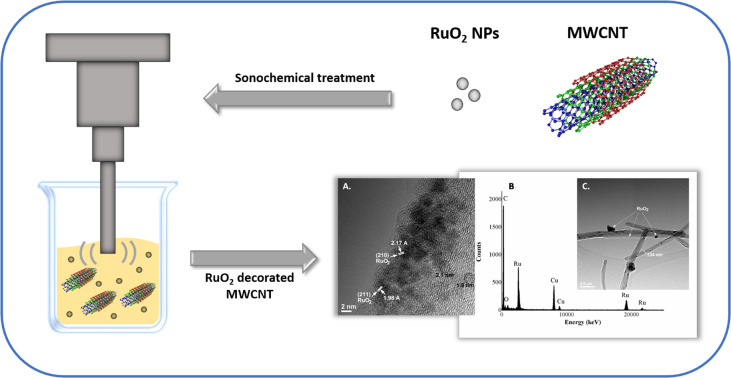

## 1. Introduction

Since their discovery in 1991 by Ijima,^1^ carbon nanotubes (CNTs) have been widely studied owing to not only their excellent and unique physical properties but also the possibility of integrating them into various composites, which show promising applications in biological materials,^2^ coatings,^3^ electronic display devices,^4^ membrane separation for water treatment, *etc.*^5,6^ In order to obtain a composite material with excellent properties, uniform distribution and good interaction between the CNTs and the polymer matrix are required.^7^ Since CNTs in their pristine form are inert and exist mainly in bundles showing a poor dispersion in both common solvents and polymer matrices, a lot of efforts were made towards the modification of the surface of CNTs to increase their solubility and processability. In general, the most common method for CNT surface modification is the chemical attachment of various functional groups, including carboxyl, hydroxyl and carbonyl groups, onto the surface of CNTs using a well-known oxidation process with concentrated acids.^8^ Another non-aggressive method involves the non-covalent functionalization of the surface of CNTs with either organic polymers or various surfactant molecules.^9^

In addition to the advantages offered by their high surface area and chemical stability, CNTs have been demonstrated as good supports for metal catalysts. The decoration of the surface of CNTs with noble metal nanoparticles has the advantages of increasing electrical or selective properties and catalytic activity with multiple uses in various fields including electrochemical sensors,^10^ fuel cells,^11^ controlled drug delivery,^12^ and catalysis.^13^ As metal nanoparticles for the surface decoration of CNTs, copper, ruthenium, titanium, and nickel were used, and CNT surface decoration was achieved by different synthesis methods, such as chemical vapor deposition, chemical reduction, electrodeposition, and electrostatic force direct assembly.^14–16^ F. H. Saboor and A. Ataei examined three different methods in order to deposit metallic nanoparticles onto the CNT surface and explain the mechanism of all methods.^17^ The decoration of CNTs with metal nanoparticles without destroying their surface was done by a liquid-phase reduction process.^18^ However, the density of coverage was low and limited by using this method. J. Lu *et al.* studied an alternative method for the decoration of the MWCNT surface with ruthenium nanoparticles, which relies on the reduction of ruthenium salt in a liquid polyol. For that, sodium dodecyl sulfate was used to activate the MWCNT surface for covering with ruthenium nanoparticles.^19^

It is well known that CNTs exhibit potent antibacterial properties against several bacteria and viruses, and hence, they have attracted significant attention for use in membrane technology over the years.^20^ The key mechanisms behind the antibacterial capabilities of CNTs are electrostatic repulsions, hydrophobic–hydrophilic interactions, physical piercing/trapping effect, and reactive oxygen species. Moreover, as a conductive metallic oxide, RuO_2_ has several uses in energy and catalysis, but there has been relatively little research done in biology, environmental preservation, and human health.^21^ Numerous recent research studies have shown that nanoparticles have potent antibacterial action against pathogens of plants and animals.

In this work, multiwalled carbon nanotubes (MWCNTs) were decorated with ruthenium dioxide (RuO_2_) nanoparticles, and this was accomplished by facile, easy and non-destructive methods such as sonochemical treatment. The surface-decorated MWCNTs (MWCNTs@RuO_2_) were morphologically and structurally investigated by transmission electron microscopy (TEM), scanning electron microscopy (SEM), X-ray photoelectron spectroscopy (XPS), X-ray diffraction (XRD), thermogravimetric analysis (TGA) and Raman spectroscopy. In contrast to other synthesis methods, the one reported herein has the advantage of being a one-step synthesis offering a lower consumption of reagents (only carbon nanotubes and ruthenium oxide are involved in the reaction) and with low energy consumption (the amount of energy for sonication is much lower than that required for advanced treatment in plasma or laser). The obtained functionalized CNTs were used as nanofillers in PEI membranes, which were further tested for antimicrobial activity using *Escherichia coli* (*E. coli*) and *Staphylococcus aureus*.

## 2. Materials and methods

Ruthenium dioxide (RuO_2_), polyetherimine and carbon nanotubes (MWCNTs) were purchased from Sigma-Aldrich. *N*,*N*′-Dimethylformamide (DMF) was purchased from Merck with analytical purity and used as received without further purification.

### 2.1. Synthesis of the decorated MWCNT with RuO_2_

The decoration of the MWCNT surface was performed *via* a sonochemical synthesis process. First, 0.08 g of CNT and 0.04 g of RuO_2_ were added to 42 ml of DMF. The mixture was then ultrasonicated for 30 minutes, filtered through a PTFE membrane with 0.22 μm pores under vacuum and dried.

### 2.2. Synthesis of the composite membrane based on PEI/MWCNT/RuO_2_

For the preparation of the homogenous polymer solution, 29 g of PEI and 150 ml of DMF (12 wt% concentration) were mixed under magnetic stirring (700 rotation speed) at 60 °C overnight. PEI/MWCNT composite membranes were obtained by adding 1 wt% of MWCNT (0.08 g) into 50 ml polymer solution and ultrasonicated (amplitude of 60%, for 30 min in an ice bath) to maintain the temperature below 5 °C. The membranes were obtained by a phase immersion method. For that, the polymer composite solution was cast onto a glass plate using a doctor blade and gently immersed in a coagulation bath, which contained 200 ml water : ethanol mixture (1 : 1). The membranes were allowed to precipitate for 2 min, taken out from the coagulation bath and thoroughly washed with distilled water to remove any residual DMF. After that, the wet membrane was dried for future characterization.

PEI/MWCNTs@RuO_2_ composite membranes were obtained using the same protocol as mentioned above (the phase immersion method) except that in the polymer solution besides 0.08 g MWCNTS, 0.04 g RuO_2_ (0.5 wt%) was added.

### 2.3. Characterization

Raman spectra were recorded using a confocal Raman microscope (Renishaw) equipped with a 473 nm excitation laser, at 0.4 mW incident power and a resolution of 2 cm^−1^.

Thermogravimetric analysis (TGA) was performed using a Q500 TA Instruments equipment, in a nitrogen atmosphere from room temperature to 900 °C at a heating rate of 10 °C min^−1^.

The elemental composition of the MWCNT and MWCNT@RuO_2_ surfaces was studied by X-ray photoelectron spectroscopy (XPS) using a K-alpha instrument from Thermo Scientific, with a monochromatic Al Kα source (1486.6 eV), at a bass pressure of 2 × 10^−9^ mbar. The charging effects were compensated by a flood gun and binding energies were calibrated by placing the C 1s peak at 284.8 eV as the internal standard. The pass energy for the survey spectra and high-resolution spectra was 200 eV and 20 eV respectively. The deconvolution of the C 1s and O 1s spectrum was done using the Gaussian–Lorentzian function.

X-ray diffraction was performed using a Panalytical X'Pert Pro diffractometer using CuKα radiation (*λ* = 1.5418 Å) from 2° to 70° (2*θ*) at a scanning rate of 1° per min.

Scanning electron microscopy (SEM) was performed using a Quanta Inspect F (FEI) instrument, with a field emission electron gun, 1.2 nm resolution and X-ray energy-dispersive spectrometer at an accelerating voltage of 30 kV.

Transmission electron microscopic (TEM) images were achieved using a Tecnai G2 F30 S-TWIN equipment provided with a 200 kV acceleration voltage emission gun.

The material's mechanical response was studied by performing the traction test using a universal mechanical tester (Instron, model 3382, USA) at a relative humidity of 45–50% and a speed of 1 mm min^−1^. The sample size was 10 cm in length and 1 cm in width. A minimum of five-seven specimens were tested for each composite materials and the average values are reported. Each experiment was carried out in triplicate (*n* = 3), and the results are presented as mean ± standard deviation.

The contact angles (*θ*) were measured on the active surface of dry membranes using a Kruss DSA100S Drop Shape Analyzer by the sessile drop method. At least five contact angle measurements were performed in different locations on the sample and the results are reported as average values. The surface free energy was estimated by the Owens, Wendt, Rabel and Kaelble (OWRK) method after measuring the contact angles for water and ethylene glycol.

The membranes' antibacterial properties were studied using *Escherichia coli* and *Staphylococcus aureus* following a protocol described in a previous study.^22^ Bacterial suspensions of *E. coli* and *S. aureus* at a concentration of 10^6^ CFU ml^−1^ were placed in contact with each sample. The bacterial suspension was incubated in polypropylene sterile tubes under the same conditions as the tested samples (*i.e.*, without coming into touch with any other material) in order to assess the positive control. After a 24 hours incubation period at 30–35 °C, PBS was added to the samples, and the aliquot was then spread out onto fresh agar plates in Petri dishes, so that the colonies could be counted and photos acquired. The samples were incubated at 30 to 35 °C for three days. The antimicrobial activity was calculated using the following equation:
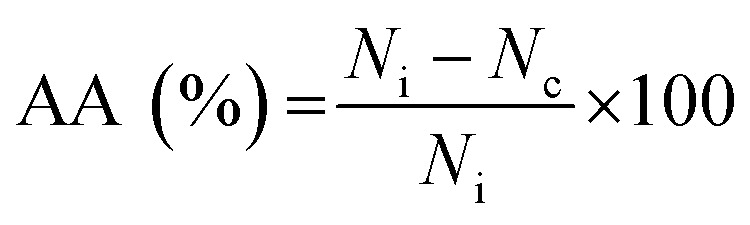
where AA is the antibacterial activity, *N*_i_ is the initial number of bacterial colonies and *N*_c_ represents the obtained number of bacterial colonies.

#### 2.3.1 Statistical analyses

The results are presented as mean ± standard deviation, and all tests were carried out in triplicate (*n* = 3). The Bonferroni post-test, one-way ANOVA, and GraphPad Prism Software 6.0 (GraphPad Software Inc., San Diego, CA, USA) were used to determine statistical significance. Differences were considered statistically significant if they were *p* < 0.05.

## 3. Results and discussions

### 3.1 Characterization of MWCNTs and MWCNT@RuO_2_

**Fig. 1:**
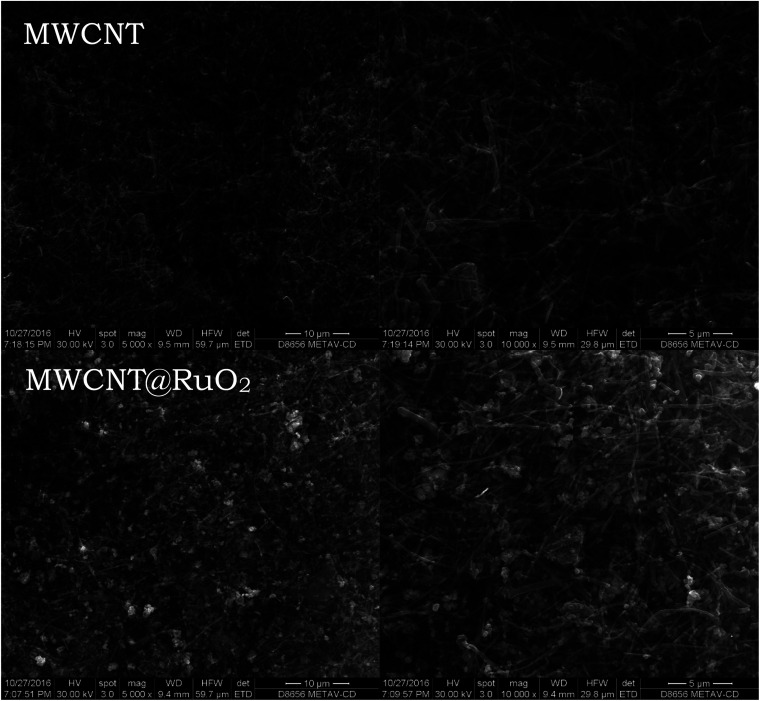
SEM images of the MWCNT and MWCNT@RuO_2_.

The morphology and general morphological features of the MWCNTs before and after decoration processes were observed by SEM. The MWCNTs were entangled randomly having a uniform diameter and length with a pointed top end of a few nanometers, as illustrated in Fig. 1. No significant difference was noticed after decoration with RuO_2_ nanoparticles, and the nanotube structure was still maintained. Furthermore, after a close examination of the images, high-contrast light spots, which are well and uniformly distributed, could be observed over the nanotube surface, which corresponds to metal oxide nanoparticles. Moreover, after the addition of the RuO_2_ nanoparticles, the surface becomes rougher than that of the pure MWCNTs.

**Fig. 2:**
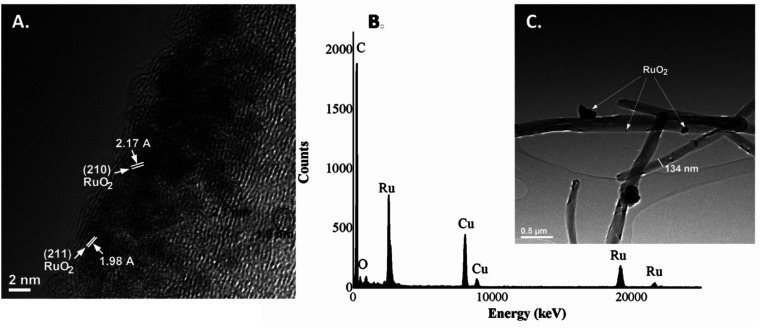
(A) HRTEM images, (B) TEM images and (C) EDX spectra of MWCNT@RuO_2_.

Due to the higher resolution, more specific structure details were provided by TEM analysis. It could be observed that the average diameter of the MWCNTs was about 134 nm and the length of the tube was several micrometers. Furthermore, considering the TEM images of the RuO_2_-decorated MWCNTs (Fig. 2), it could be found that the surface of MWCNTs was uniformly covered with very fine, small RuO_2_ nanoparticles with an average size of about 2 nm.^23^ Moreover, the lattice fingers of the nanotube could be clearly seen in the image. In addition, RuO_2_ nanoparticles display a narrow size distribution along the walls of the tubes being assembled into a monolayer on the MWCNT surface (Fig. 2A). Since no free RuO_2_ nanoparticles were observed in the background of the TEM images, this means that all the nanoparticles were completely used by the MWCNTs. The crystallographic planes are presented at 210 and 211 lattices, which correspond to interplanar distances of 2.17 A and 1.98 A respectively.^24,25^ These results are in agreement with the XRD data presented below.

The presence of the RuO_2_ on the MWCNT surface was also confirmed in the EDS spectrum (Fig. 2B), *i.e.*, the elemental analysis showing the presence of C, O and Ru as the main components within the sample. The minor amount of Cu element within the spectrum is the background from the TEM support grid.

**Fig. 3:**
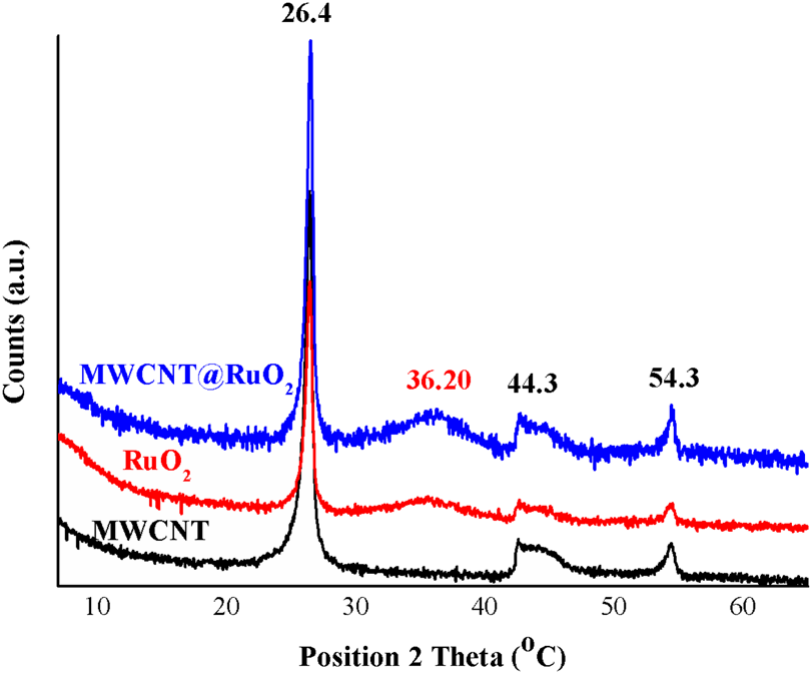
XRD spectra of MWCNT and MWCNT@RuO_2_.

The XRD diffractograms of the MWCNT and MWCNT decorated with RuO_2_ nanoparticles are shown in Fig. 3. Three distinct peaks, which correspond to the graphite structure of MWCNT, at 26.4°, 44.3° and 54.3° assigned to the (002), (101) and (004) planes of CNTs, were detected in all diffractograms.^26^ Owing to the fact that a low amount of the metal is anchored onto the MWCNT surface and also to the overlapping with the carbon peaks, the characteristic peaks for RuO_2_ nanoparticles were not clearly visible in the MWCNT@RuO_2_ pattern. Conversely, after a close examination of the MWCNT@RuO_2_ spectrum, a wide bump could be observed at 36.2° (101), and this is assigned to the tetragonal phase of ruthenium, which confirms the existence of metallic nanoparticles according to JCPDS-21-1172.^27,28^ These results combined with the EDS analysis presented above lead to the conclusion that the MWCNT surface is successfully decorated with RuO_2_ nanoparticles.

**Fig. 4:**
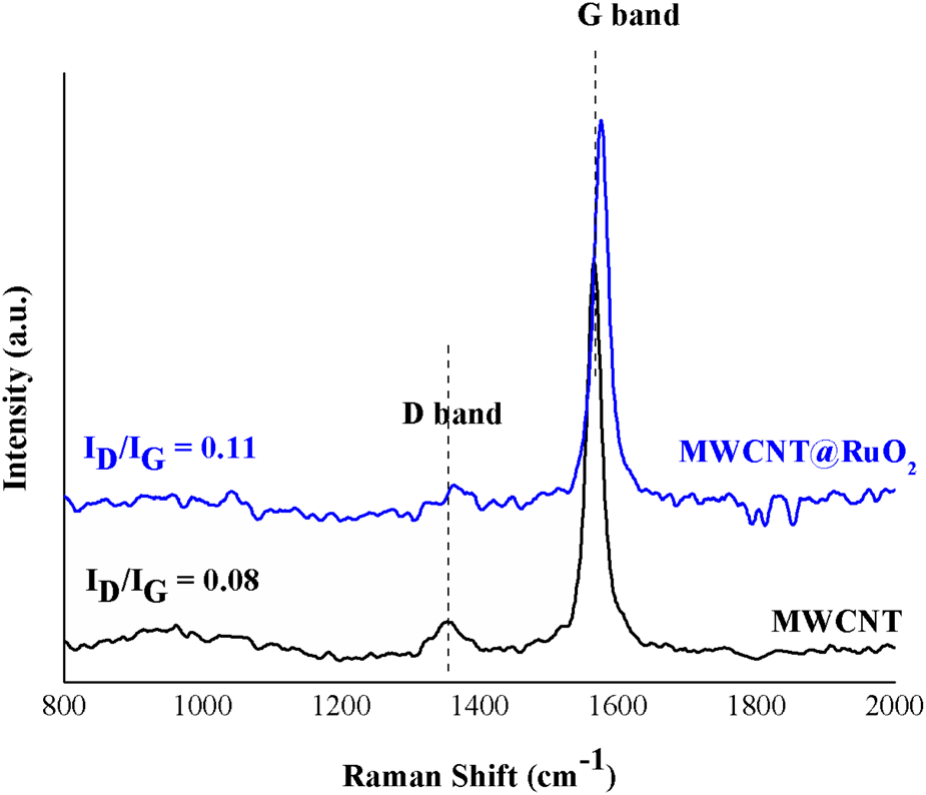
Raman spectra of MWCNT and MWCNT@RuO_2_.

Furthermore, in order to study the interaction of the RuO_2_ nanoparticles on the MWCNT surface, Raman spectra were recorded for both pure MWCNTs and MWCNTs@RuO_2_. The spectra were acquired over a Raman shift range of 200–3600 cm^−1^. According to Fig. 4, both samples showed two characteristic peaks at 1350 cm^−1^ and 1564 cm^−1^ assigned to sp^3^ (D band)- and sp^2^-hybridized carbons (G band), which confirm the existence of disordered graphite and ordered-state graphite.^29^ For more information about the decoration process, the intensity ratios of the D and G band (*I*_D_/*I*_G_) were calculated for all the samples,^30^ and this is an important method for the diagnosis of defect concentration. It was observed that the *I*_D_/*I*_G_ ratio of MWCNT@RuO_2_ was slightly higher than that of pure MWCNT (0.11 to 0.8), which can lead to the conclusion that the RuO_2_ nanoparticles were decorated onto the MWCNT surface. Moreover, the strong interaction of RuO_2_ onto the surface of MWCJNT was also shown by the positive shifts of the D (1350 to 1362 cm^−1^) and G (1564 to 1575 cm^−1^) bands.

**Fig. 5:**
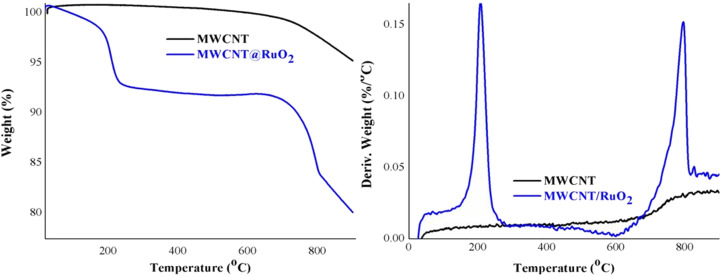
TGA and DTG curves of MWCNT and MWCNT@RuO_2_.

Additional information about the decoration of the MWCNT with RuO_2_ was obtained from the thermogravimetric analysis displayed in Fig. 5. Pure MWCNTs are stable and registered one degradation step with a little low weight of around 5%, which could be assigned to the combustion of the C–C bond from the CNT structure.^31^ By comparison, MWCNT@RuO_2_ exhibited two distinct degradation steps at 100–250 °C and 600–850 °C respectively with a weight loss of nearly 20%. The slight first decomposition step corresponds to the removal of adsorbed water and crystal water, while the second step, larger, is attributed to the residue of RuO_2_ and indicates the presence of remnant RuO_2_.^32^ Considering this, the amount of RuO_2_ in the MWCNT@RuO_2_ samples was calculated based on the TG results and it is about 15%.

**Fig. 6:**
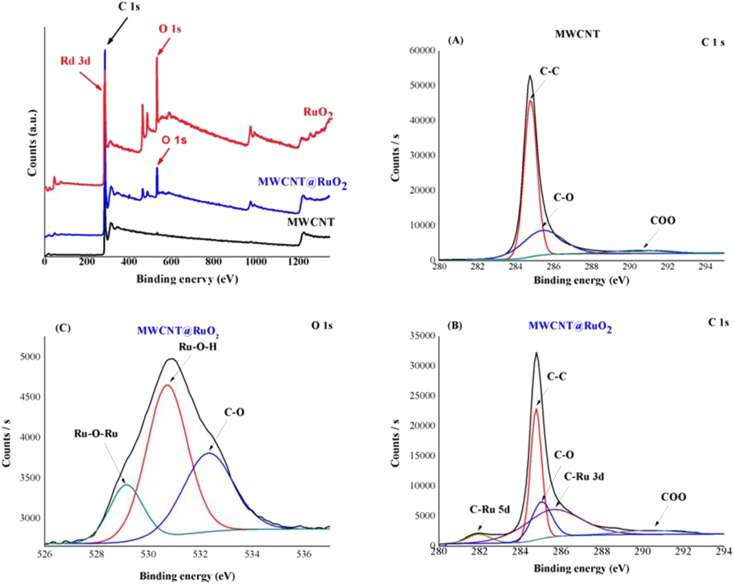
XPS survey spectra of MWCNTs before and after decoration with RuO_2_: high-resolution C 1s spectra of MWCNT (A) and MWCNT@RuO_2_ (B); and high-resolution O 1s spectra of MWCNT@RuO_2_ (C).

The chemical composition of MWCNT@RuO_2_ was investigated by XPS analysis. As observed in Fig. 6, the XPS survey spectrum of the pure MWCNT shows the two expected elements C and O at 284.8 eV and 532.6 eV respectively, and their atomic percentage was 99.13% and 0.87%. A new peak could be detected in the XPS survey spectrum of MWCNT@RuO_2_ at 284.3 eV with an atomic percentage of 5.19%, which corresponds to Ru 3d.

For more information regarding the decoration of the MWCNT surface with RuO_2_ nanoparticles, the C 1s and O 1s XPS spectra were recorded. Since the Ru 3d_3_ spectrum is generally overlapped by C 1s spectrum, for the identification of the electronic states of Ru, the Ru 3d_5_ spectrum is used.^33^ Thus, the C 1s spectra of MWCNTs could be deconvoluted into three peaks at 284.8, 285.4 and 290.4 eV assigned to the C–C/C=C, COO groups respectively.^34^ Conversely, the C 1s spectrum in the case of MWCNT@RuO_2_ shows the presence of two new peaks at 281.9 and 285.03 eV, which correspond to the C–Ru bond, confirming the successful decoration of MWCNT surface with RuO_2_. Similarly, the O 1s deconvolution spectrum of MWCNT@RuO_2_ exhibits three peaks located at 529.14, 530.7 and 532.3 eV attributed to Ru–O–Ru, Ru–O–H and C–O. Interestingly, a slight shift in the C 1s and O 1s peaks of MWCNTs@RuO_2_ compared with MWCNTs confirmed the strong interaction between MWCNT and RuO_2_ nanoparticles. The obtained results are in accordance with the data reported in the literature^29^ and are another proof for the decoration of the MWCNT surface with metal nanoparticles.

### 3.2 Characterization of composite membranes based on PEI/MWCNT/RuO_2_

The interest in PEI-reinforced composites comes from their potential application as membrane materials. Thus, the modified MWCTs were further introduced into a PEI matrix and different composite membranes were obtained. The membranes were synthesized using a versatile method such as the phase-inversion method. For all types of membrane processes, it is possible to obtain polymeric membranes by starting with a polymer solution and a non-solvent and adjusting the coagulation, temperature, and concentration of a polymer solution with a non-solvent.

**Fig. 7:**
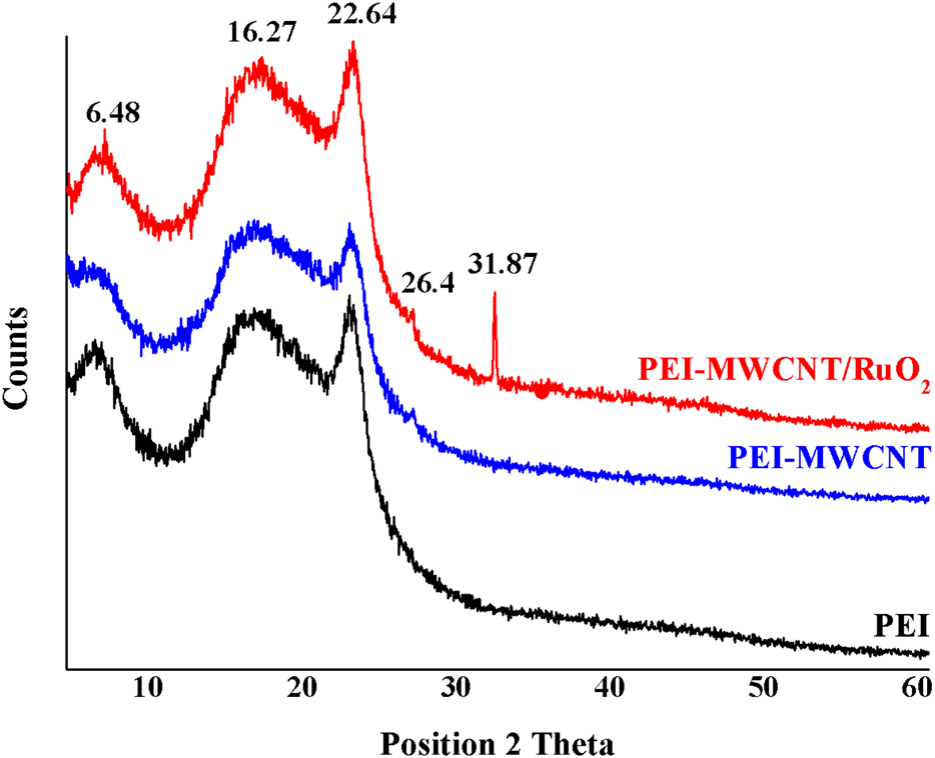
XRD spectra of PEI, PEI-MWCNT and PEI-MWCNT@RuO_2_.

The XRD analysis shows the modification of the polymer crystallinity induced by the incorporation of both MWCNT and MWCNT@RuO_2_ and also the possible changes of the characteristic diffraction peaks. According to Fig. 7, PEI membranes display two broad diffraction peaks centered at 2*θ* = 5.72 and 16.22° and a sharp peak at 2*θ* = 22.39°. After the incorporation of MWCNTs and MWCNTs@RuO_2_ within the polymer matrix, only slight changes were observed in terms of the crystalline nature of the polymer. Moreover, a new diffraction peak at 2*θ* = 26.4° assigned to the MWCNTs could be observed in the case of PEI-MWCNT, and another one at 2*θ* = 31.87° attributed to RuO_2_ was observed in the case of PEI-MWCNT@RuO_2_.

**Fig. 8:**
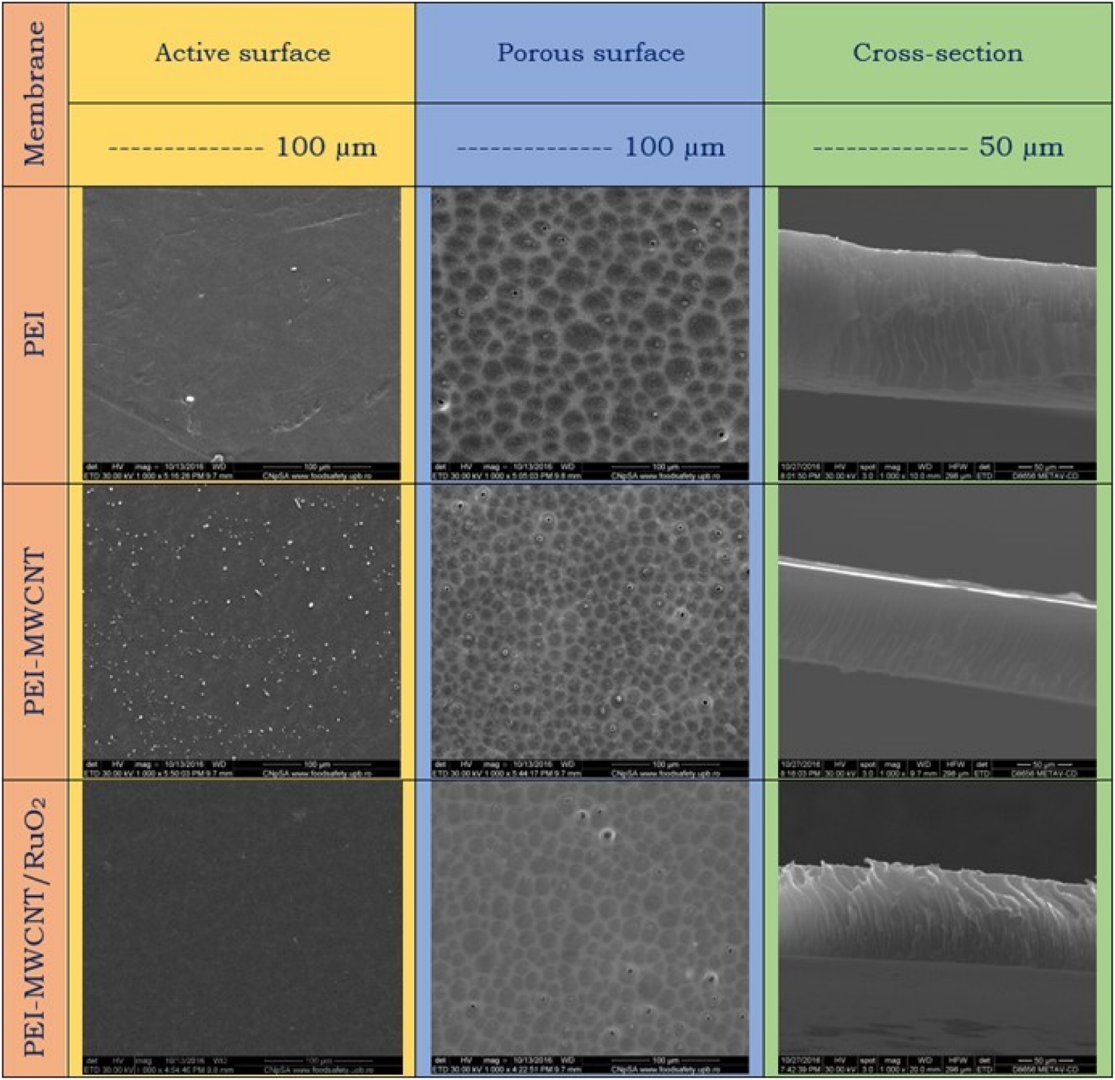
SEM images of the PEI and composite membranes.

Important details about the morphology of the produced membranes are revealed by the SEM images (Fig. 8), where the pictures showed the characteristic asymmetric structure of the membranes obtained by the phase immersion method. The asymmetric structure of the membrane consists of two distinct layers; an active layer (the dense surface) followed by macrovoids and the porous layer (support surface). The active surface of the pure PEI membrane shows a relatively smooth morphology, which tends to be more rough by the addition of the reinforcing agent within the polymer matrix. This surface became a bit smooth with MWCNTs@RuO_2_ addition. Moreover, according to the SEM images, the porous surface is quite uniform and homogenous, and has a rather compact shape for all the samples. Pure PEI membrane pores have a round shape and roughly equal diameter. However, after the addition of MWCNTs within the polymer matrices, a modification of the morphology, from a qualitative point of view, could be observed in the images. It looks like the PEI-MWCNT composite membrane's pore distribution increased while its diameter decreased due to a crosslinking effect that seems to be connected to weak chemical interactions between the polymer and filler. This can be explained by the capacity of MWCNTs to promote a more compact arrangement of the entire mass by slowing down the coagulation bath in-diffusion and solvent out-diffusion. Similar findings were published by Badawi *et al.*, who discovered that the functionalized carbon nanotubes with a –COOH group might delay phase inversion by forming H bonds with the nonsolvent molecules (deionized water) [N. El Badawi, A. R. Ramadan, A. M. K. Esawi and M. El-Morsi, *Desalination*, 2014, 344, 79]. Additionally, it appeared that CNTs blocked some of the pores due to their distribution across the polymer matrix. By contrasting the SEM micrographs of the PEI-MWCNT membranes and the PEI-MWCNT@RuO_2_ membranes, it can be seen that the pore distribution for the decorated MWCNT membrane reduces as the diameter of the carbon nanotubes grows. Furthermore, considering the cross-section of the membranes, pure PEI and PEI-MWCNT@RuO_2_ composite membranes showed comparable morphology, with macrovoids oriented perpendicular to the membrane surface; in contrast, the macrovoids on the membrane surface of the PEI-MWCNTs composite membrane tended to be oblique.

In order to assess the impact of both MWCNTs and MWCNTs decorated with RuO_2_ nanoparticles on the mechanical properties of the PEI, Young's modulus and tensile stress were calculated for all the samples. The obtained results are presented in Table 1. According to the obtained results, in the case of the PEI-MWCNT composite membranes, it can be said that both the tensile stress and Young's modulus increase compared with pure PEI. This enhancement of the tensile strength is due to the dispersion of the MWCNTs within the polymer matrix and also confirms the formation of some physical binding between the two compounds. Similar results were also obtained in the case of sulfonated poly(ether ketone)/polyetherimide composite membranes with low amounts of single-walled nanotubes.^35^ Moreover, an increase in the tensile stress could be further observed in the case of the PEI-MWCNTs@RuO_2_ composite membranes, indicating better ductility and decreased brittleness. This is because, in contrast to MWCNTs and PEI, the interface between RuO_2_ nanoparticles and PEI-MWCNTs may have a stronger binding due to hydrogen bonds and electrostatic interactions. This improved bonding can avoid separation when subjected to high strain. Conversely, a decrease in Young's modulus has been recorded in this case. One of the reasons for this change is that RuO_2_ nanoparticles are somewhat weaker and less stiff than MWCNTs, mostly as a result of their ionic bonding. It is possible for RuO_2_ particles to break apart the linked structure of MWCNTs contained in the PEI matrix. This disturbance may result in less effective load distribution and stress transfer between the PEI polymer chains and the carbon nanotubes.^36^

**Table 1:** Young's modulus and tensile stress of PEI, PEI-MWCNTs and PEI-MWCNTs@RuO_2_

Sample name	Young's modulus (MPa)	Tensile stress (MPa)
PEI	8.1 ± 0.43	2.40 ± 0.31
PEI-MWCNTs	11.2 ± 1.67	2.55 ± 0.36
PEI-MWCNTs@RuO_2_	7.9 ± 0.79	2.85 ± 0.38

The hydrophilic character of the membranes is also an important aspect that needs to be taken into consideration. Therefore, contact angle measurements were performed to evaluate the hydrophilicity of the composite membranes, and the data are reported in Table 2. According to the obtained results, an increased hydrophilicity for both composite membranes was registered compared with pure PEI membranes. The most hydrophilic membrane is PEI-MWCNTs. This enhancement is attributed to the effect on the membrane surface properties given by the hydrophilic character of the MWCNTs offering at the same time an improved fouling resistance.^37^ However, the rough structure derived from the MWCNTs can improve the membrane's wettability, as observed in SEM images. Hydrophilic surfaces can be formed more easily by increasing surface roughness because the membrane surface can solidify some water molecules into its hierarchical structure. The same observation was seen by Yahong Sun *et al.* in their study where they decorated the SWCNT surface with TiO_2_ in order to develop superwetting composite membranes for highly efficient oil-in-water emulsion separation.^38^ Moreover, PEI-MWCNTs@RuO_2_ composite membranes exhibit a decrease in hydrophilicity compared with PEI-MWCNTs because the RuO_2_ nanoparticles modify the surface of the MWCNTs, including their electric nature. We also computed the samples' surface free energy using the contact angle values for distilled water and ethylene glycol (EG). It was discovered that PEI membranes have a surface free energy of 34.47 mN m^−1^, which rises when the nanofiller is added. As would be expected following the addition of the MWCNTs and MWCNTs@RuO_2_, the data in Table 2 seem to indicate that the increase in surface free energy is caused by a rise in the polar portion compared with pure PEI membrane.

**Table 2:** Contact angle data for PEI, PEI-MWCNTs, and PEI-MWCNTs@RuO_2_ composite membranes

Sample	Water (*θ*)	EG (*θ*)	Surface free energy (mN m^−1^)	Polar part (mN m^−1^)	Dispersive part (mN m^−1^)
PEI	84 ± 2	55 ± 1	34.47 ± 7	30.35	4.1
PEI-MWCNTs	61 ± 3	56 ± 3	47.47 ± 10	46.07	1.4
PEI-MWCNTs@RuO_2_	74 ± 0.3	34 ± 2	46.60 ± 3	41.18	5.4

The antimicrobial activity of the MWCNTs is well known but most of the studies are focused more on metallic nanoparticle-decorated CNTs as efficient antimicrobial agents especially Ag-decorated CNTs. In our study, the antimicrobial activity of the PEI was also tested and we observed an increased antimicrobial activity up to 75% ± 5% in the case of *E. coli* for PEI-MWCNT composite membranes and PEI-MWCNTs@RuO_2_ respectively (three different samples were tested for statistical analysis). Better results were observed in the case of *Staphylococcus aureus*, where antimicrobial efficiencies of 90 ± 5% have been registered for the composite membranes. Cell death is caused by the structural changes induced *via* the static relationship amongst the negative-charged cells of bacteria and the positively charged nanoparticles that are necessary for the nanoparticles to operate as bactericidal agents.^39^

## 4 Conclusions

In summary, MWCNTs decorated with RuO_2_ nanoparticles were prepared using an easy and efficient sonochemical method. The uniform decoration of the MWCNT surface with metallic nanoparticles was confirmed by TEM analysis, where a narrow size distribution of the RuO_2_ nanoparticles along the CNT wall could be observed. Moreover, according to the SEM images, the structure of MWCNTs was not been affected after the sonochemical process, the nanoparticles being well and uniformly distributed among CNTs length. Moreover, the presence of RuO_2_ nanoparticles had been proved by EDX and XRD analysis. Raman spectroscopy and XPS analysis further revealed the strong interaction between the two compounds. The percentage of the RuO_2_ nanoparticles onto the MWCNT surface was calculated considering the weight loss from TG analysis and 15%RuO_2_ has been recorded. The composite membranes were synthesized by a phase-inversion method using a concentration of 1 wt% MWCNTs and 1 wt% MWCNTs@RuO_2_ as nanofillers. According to the SEM images, the composite membranes show an increase in the pore distribution and a decrease in the pore diameter compared with pure PEI. Moreover, after incorporation of the nanofiller within the polymer matrices both the tensile stress and Young's modulus increase in the case of PEI-MWCNT composite membranes. However, in the case of PEI-MWCNTs@RuO_2_ composite membranes, Young's modulus decreased due to the presence of metallic nanoparticles around the MWCNT length. Considering the surface properties of the composite membranes, the contact angle measurements exhibit an increase in the hydrophilic character for those samples after the incorporation of the nanofiller within the polymer matrices. Moreover, the composite membranes exhibit good antimicrobial activity for both *E. coli* and *Staphylococcus aureus*.

## Data availability

The authors confirm that the data supporting the findings of this study are available within the article.

## Conflicts of interest

The authors declare no competing interests.
